# Ammonia gas sensors based on In_2_O_3_/PANI hetero-nanofibers operating at room temperature

**DOI:** 10.3762/bjnano.7.122

**Published:** 2016-09-19

**Authors:** Qingxin Nie, Zengyuan Pang, Hangyi Lu, Yibing Cai, Qufu Wei

**Affiliations:** 1 Key Laboratory of Eco-Textiles, Ministry of Education, Jiangnan University, 214122 Wuxi, Jiangsu, China

**Keywords:** ammonia (NH_3_), electrospinning, gas sensor, indium(III) oxide (In_2_O_3_), polyaniline (PANI)

## Abstract

Indium nitrate/polyvinyl pyrrolidone (In(NO_3_)_3_/PVP) composite nanofibers were synthesized via electrospinning, and then hollow structure indium oxide (In_2_O_3_) nanofibers were obtained through calcination with PVP as template material. In situ polymerization was used to prepare indium oxide/polyaniline (In_2_O_3_/PANI) composite nanofibers with different mass ratios of In_2_O_3_ to aniline. The structure and morphology of In(NO_3_)_3_/PVP, In_2_O_3_/PANI composite nanofibers and pure PANI were investigated by scanning electron microscopy (SEM), Fourier transform infrared spectroscopy (FTIR), X-ray diffraction (XRD), transmission electron microscopy (TEM) and current–voltage (*I*–*V*) measurements. The gas sensing properties of these materials towards NH_3_ vapor (100 to 1000 ppm) were measured at room temperature. The results revealed that the gas sensing abilities of In_2_O_3_/PANI composite nanofibers were better than pure PANI. In addition, the mass ratio of In_2_O_3_ to aniline and the p–n heterostructure between In_2_O_3_ and PANI influences the sensing performance of the In_2_O_3_/PANI composite nanofibers. In this paper, In_2_O_3_/PANI composite nanofibers with a mass ratio of 1:2 exhibited the highest response values, excellent selectivity, good repeatability and reversibility.

## Introduction

With the development of modern industry, environmental pollution in the form of air pollution, water pollution and soil pollution has become ever more serious [1]. With regard to this, considerable attention has been paid to air pollution. Ammonia (NH_3_), as a highly toxic gas, can be emitted by natural and industrial sources and threaten human health [2–4]. NH_3_ at concentrations of 50 ppm may irritate the human respiratory system, skin and eyes [4]. Higher concentrations of NH_3_ will cause blindness, seizures, lung disease and even death [5–7]. So, there is an urgent need to develop a kind of gas sensor with high sensitivity and selectivity to detect NH_3_ at room temperature.

Metal oxide semiconductors can be applied as sensing materials for monitoring NH_3_. Ammonia sensors based on In_2_O_3_ [8], TiO_2_ [9], SnO_2_ [10], ZnO [11] and WO_3_ [12] have been reported. Indium oxide (In_2_O_3_) is an n-type semiconductor with a band gap of approximately 3.55–3.75 eV, which has been widely used due to its excellent electrical and optical properties. In_2_O_3_ also exhibits sensitivity to various vapors and gases, such as NO_2_ [13], CO [14], H_2_ [15], acetone [16] and formaldehyde [17]. However, for most metal oxides, there is the drawback of a required high operation temperature, about 300 °C, which will increase the energy consumption [18]. Compared with metal oxides, sensors based on conducting polymers show low power consumption and can be operated at room temperature. In addition, they exhibit a large specific area, small size and low weight, and they are easy to integrate with existing electronics [19–20]. Because of the environmental stability, easy synthesis and reversible doping behavior, polyaniline (PANI), as one of the most commonly used conducting polymers has received considerable attention. However, the sensitivity of PANI remains to be improved [21–22]. To conquer the limitations mentioned above, the combination of metal oxide and conducting polymers have been developed as an effective way to achieve enhanced performance [21,23–27].

In this paper, In_2_O_3_/PANI composite nanofibers were prepared by the combination of electrospinning technique, calcination method and in situ polymerization. This study presents the improved response capabilities of gas sensors based on In_2_O_3_/PANI composite nanofibers, which were synthesized with different ratios between In_2_O_3_ and aniline during the in situ polymerization. All sensors were tested at room temperature in a concentration range of NH_3_ from 100 to 1000 ppm.

## Experimental

### Materials

Polyvinylpyrrolidone-K90 (PVP-K90, *M*_w_ = 1.3 × 10^6^ g/mol) was purchased from Bo Di Industrial Co. Ltd of Tianjin. Indium nitrate hydrate, *N*,*N*-dimethylformamide (DMF), ethyl alcohol, aniline monomer (An), ammonium persulfate (APS), hydrochloric acid (HCl, 37%), ammonium hydroxide (NH_4_OH) and *m*-cresol were obtained from Sinopharm Chemical Reagent Co., Ltd. (Shanghai, China). All chemicals and reagents were used as received, expect for aniline monomer. Distilled aniline monomer and deionized water were used in this study.

### Preparation of hollow In_2_O_3_ nanofibers

The In(NO_3_)_3_/PVP composite nanofibers were fabricated via single-nozzle electrospinning. In(NO_3_)_3_·4.5H_2_O (1.059 g) and PVP (3.529 g) were added into 10 mL ethyl alcohol and 10 mL DMF. The mixture was stirred at 65 °C until all the solutes were fully dissolved. The precursor solution was poured into the syringe for electrospinning. The parameters of the electrospinning were: a needle-to-collector distance of 16 cm, a voltage of 16 kV, and a feed rate of 0.5 mL/h. Then the In_2_O_3_ nanofibers were synthesized by annealing the precursor composite nanofibers at 800 °C for 3 h after heating from room temperature at a rate of 0.5 °C/min.

### Preparation of In_2_O_3_/PANI composite nanofibers

**Figure 1:**
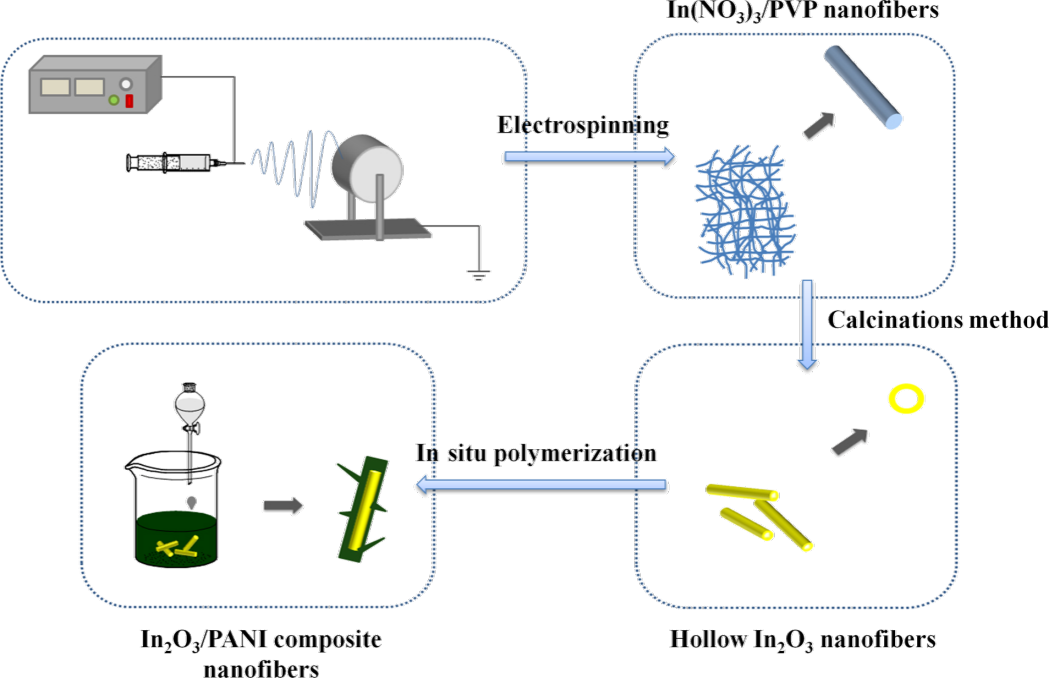
Schematic of the preparation of In_2_O_3_/PANI composite nanofibers.

Firstly, 0.1 g In_2_O_3_ nanofibers, which had been ground in an agate mortar, were added into 200 mL 1.2 mol/L HCl solution with ultrasonication treatment. Then a certain amount of aniline in HCl solution was added to the above suspension. After that, 30 mL 1.2 mol/L HCl solution containing APS was slowly dripped into the suspension to initiate the polymerization. The mass ratios of In_2_O_3_ nanofibers to aniline were 1:1, 1:2 and 1:4. The molar ratio of aniline to APS was 1:1. The in situ polymerization of aniline was carried out in an ice bath at 0–5 °C. The reaction lasted for 5 h. The suspension was taken out and left for 30 min, and then washed with deionized water and centrifuged for 5 min. At last, the composite nanofibers were filtered and dried in vacuum at 50 °C for 48 h. The schematic of the preparation of In_2_O_3_/PANI composite nanofibers is illustrated in Figure 1.

### Fabrication of In_2_O_3_/PANI gas sensors

The ground In_2_O_3_/PANI nanofibers and pure PANI were mixed with *m*-cresol to form pastes, in which the weight ratio of In_2_O_3_/PANI or PANI to *m*-cresol was 1:10. Each paste was coated onto interdigital electrodes to construct a sensing film and dried at 55 °C for 2 h in air. Four thin film sensors with different mass ratios of In_2_O_3_ to aniline (0, 1:1, 1:2 and 1:4) were prepared. Correspondingly, the four sensors were denoted as PANI sensor, In_2_O_3_/PANI-1 nanofibers sensor, In_2_O_3_/PANI-2 nanofibers sensor and In_2_O_3_/PANI-3 nanofibers sensor.

### Structural characterization and gas sensing test

The crystal structure of In_2_O_3_ nanofibers was characterized by X-ray diffraction (XRD; D8, Bruker AXS, Germany) in a 2θ region of 3–90° with Cu Kα radiation. The morphologies and structures of the In_2_O_3_ nanofibers, PANI and PANI/In_2_O_3_ composite nanofibers were examined by field emission scanning electron microscopy (FESEM, S-4800 and SU-1510, Hitachi, Tokyo, Japan), transmission electron microscopy (TEM; JEM-2100HR, JEOL), and Fourier transform infrared (FTIR) spectroscopy in the range of 4000–400 cm^−1^ with a 4 cm^−1^ spectral resolution (NEXUS 470 spectrometer, Nicolet, Madison, WI, USA). *I*–*V* measurements were carried out on a CHI 660E electrochemical workstation (CH Instruments, Shanghai, China) with a three-electrode system.

The gas sensing performance of the prepared In_2_O_3_/PANI nanofibers was measured using a custom-built static state gas sensing test system at room temperature (25 ± 1 °C) with a relative humidity of 60 ± 1%. During the gas measurement, the aqueous ammonia was injected into the test chamber using a syringe through a rubber plug. The volume of ammonia injected into chamber were 1.3468 μL, 4.0404 μL, 6.734 μL, 10.7744 μL and 13.468 μL resulting in ammonia vapor with concentrations of 100, 300, 500, 800 and 1000 ppm, respectively. The gas response value (*S*) is defined as a ratio of (*R_i_* − *R*_0_)/*R*_0_, in which *R_i_* and *R*_0_ are the resistance of the sensor in testing gas and air, respectively. Each result was the average value of five tests.

## Results and Discussion

### Materials characterization

**Figure 2:**
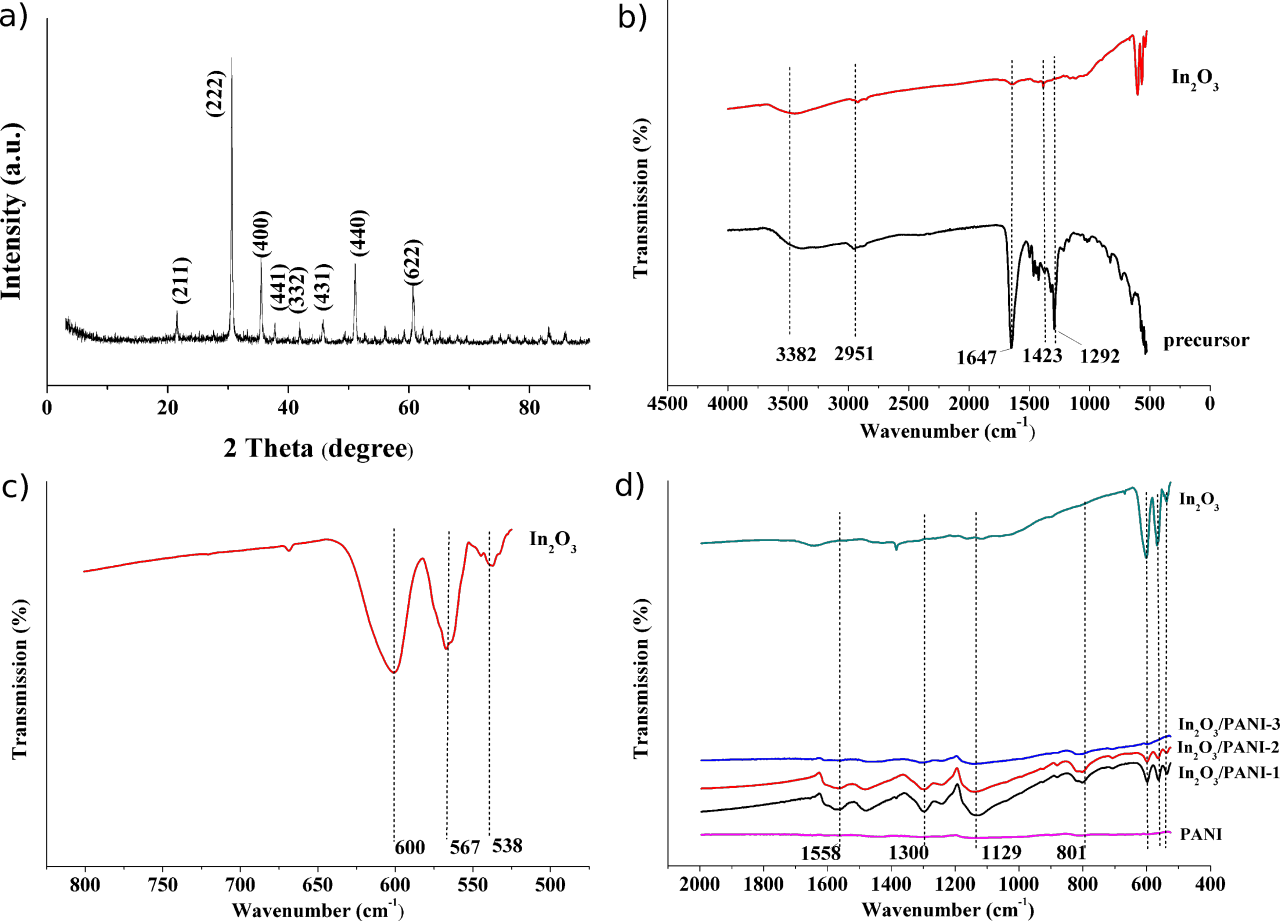
a) XRD pattern of In_2_O_3_ nanofibers. FTIR spectra of b) In(NO_3_)_3_/PVP composite nanofibers and In_2_O_3_ nanofibers, c) In_2_O_3_ nanofibers, and d) In_2_O_3_ nanofibers and In_2_O_3_/PANI composite nanofibers.

The XRD patterns of the nanofibers obtained by annealing In(NO_3_)_3_/PVP composite nanofibers at 800 °C are shown in Figure 2a. It can be seen that the crystal phase of the material was In_2_O_3_, and the diffraction peak of 30.56° was indexed to the (222) crystal plane of the cubic structure of In_2_O_3_. This result confirmed that the final product of calcination was In_2_O_3_.

The chemical structure of the precursor nanofibers, In_2_O_3_ nanofibers and In_2_O_3_/PANI nanofibers were analyzed by FTIR. As shown in Figure 2b, the FTIR spectrum of In(NO_3_)_3_/PVP composite nanofibers exhibits a broad characteristic band around 3382 cm^−1^, which is related to the O–H stretching vibration. It could be the result of absorbing moisture from air. The –CH_2_ stretching vibration and bending vibration of PVP are attributed to the peaks of around 2951 cm^−1^ and 1423 cm^−1^. The peaks at 1647 cm^−1^ and 1292 cm^−1^ were assigned to the C=O stretching vibration and C≡N antisymmetrical stretching vibration, respectively, in the ring skeleton of PVP. But the characteristic peaks of In(NO_3_)_3_ could not be found in the FTIR spectra. In the spectrum of In_2_O_3_ nanofibers (Figure 2c), the characteristic peaks of PVP have almost vanished. Instead, peaks around 600 cm^−1^, 567 cm^−1^ and 538 cm^−1^ appeared, which are associated with the cubic bixbyite-type structure of In_2_O_3_. The results indicates that PVP was resolved and In(NO_3_)_3_ was converted into In_2_O_3_ during annealing.

Figure 2d presents the FTIR spectra of In_2_O_3_, PANI and In_2_O_3_/PANI nanofibers. The characteristic peaks around 1558 cm^−1^ originating from C=C stretching vibration in the quinoid ring of PANI can be seen. The characteristic bands of 1300 cm^−1^ and 1116 cm^−1^ were attributed to the C–N stretching vibration in the benzenoid ring and the bending vibration plane of C–H bonds in the quinoid ring, respectively. By comparison, the characteristic peaks of In_2_O_3_ also exist in the spectrum of In_2_O_3_/PANI nanofibers. This demonstrated that PANI was coated on the surface of the In_2_O_3_ nanofibers by in situ polymerization. But for In_2_O_3_/PANI-3 nanofibers, the peaks of In_2_O_3_ and PANI showed a significantly decrease which may be caused by the excess of PANI covering.

**Figure 3:**
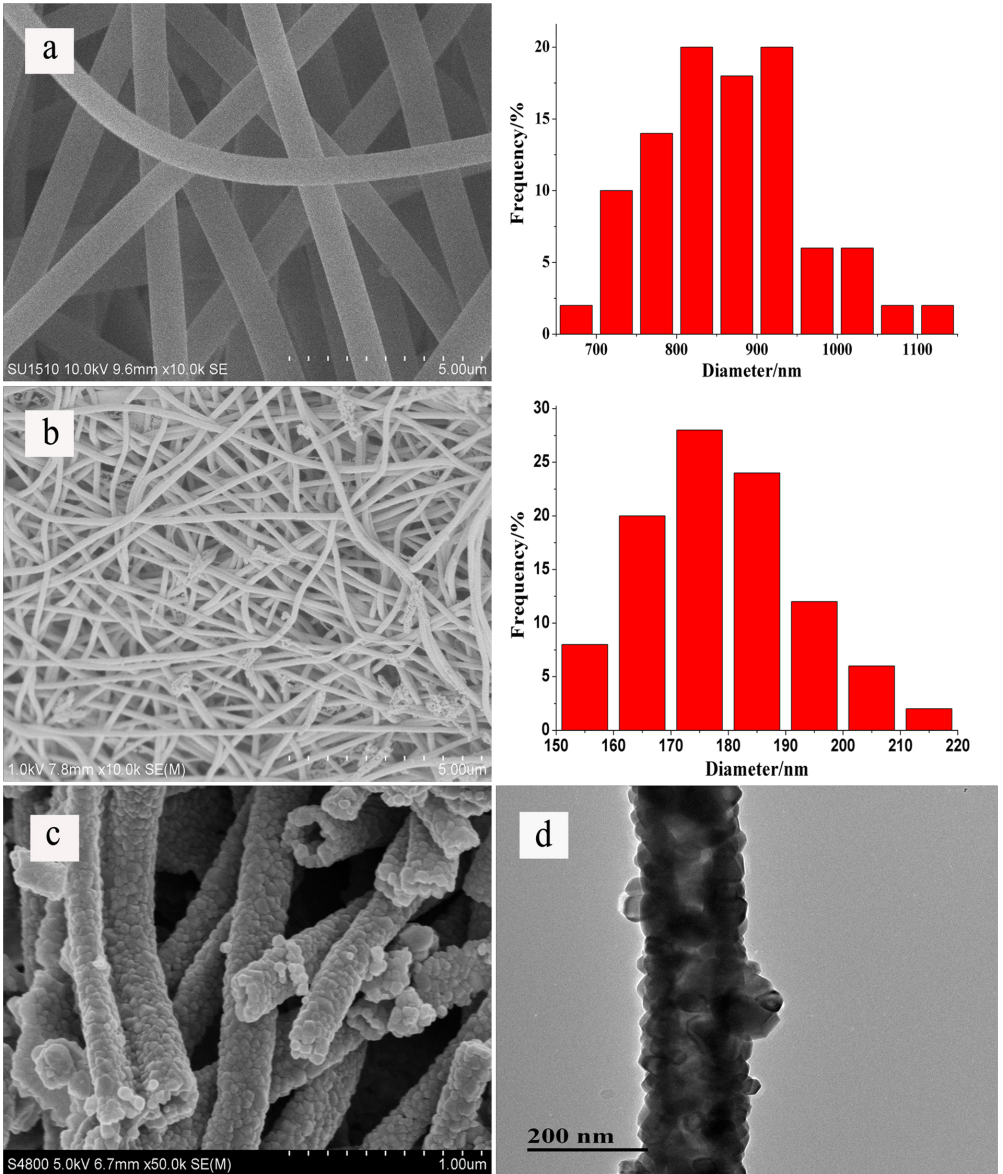
SEM images of (a) In(NO_3_)_3_/PVP composite nanofibers (with diameter distributions), (b) In_2_O_3_ nanofibers (with diameter distributions), (c) cross-section of In_2_O_3_ nanofibers. (d) TEM image of In_2_O_3_ nanofibers.

Figure 3a,b shows SEM images of In(NO_3_)_3_/PVP composite nanofibers and In_2_O_3_ nanofibers, respectively. From Figure 3a, it can be seen that the surface of In(NO_3_)_3_/PVP composite nanofibers was relatively smooth and no beads and droplets appeared. The diameter distribution of In(NO_3_)_3_/PVP nanofibers were mostly in the range of 700–1000 nm. The In_2_O_3_ nanofibers were relatively uniform with diameters of 150–220 nm. These results show that the In_2_O_3_ nanofibers were much rougher and smaller than the precursor nanofibers. In addition, it shows that some nanofibers adhered together in Figure 3b, which is not the case in Figure 3a. It is possible that the solvent was not completely volatilized from the precursor nanofibers membrane. The residual solvent may have re-dissolved the precursor nanofibers and then the dissolved nanofibers were connected along each other during the calcinations. As the calcination temperature increased, the residual solvent started to volatilize and the In_2_O_3_ nanofibers were gradually formed, but the bonded nanofibers would not segregate during this process, leading to some nanofibers connected along each other.

The cross-sectional image and the TEM image show the detailed structure of In_2_O_3_ nanofibers. In Figure 3c and 3d, it is confirmed that the In_2_O_3_ nanofibers consited of small grains and the hollow structure can be clearly observed. The hollow structure of In_2_O_3_ nanofibers was synthesized by a template-assisted method. PVP as the supporting material of precursor nanofibers was decomposed during the annealing process, and the In(NO_3_)_3_ was transformed into crystalline In_2_O_3_. During calcination, PVP decomposed and gases diffused from the interior to the exterior of the composite nanofibers, leading to crystalline In_2_O_3_ grains constantly moving and tending to be regularly arrayed [16]. As a result, hollow structure of In_2_O_3_ nanofibers was formed during the annealing process.

**Figure 4:**
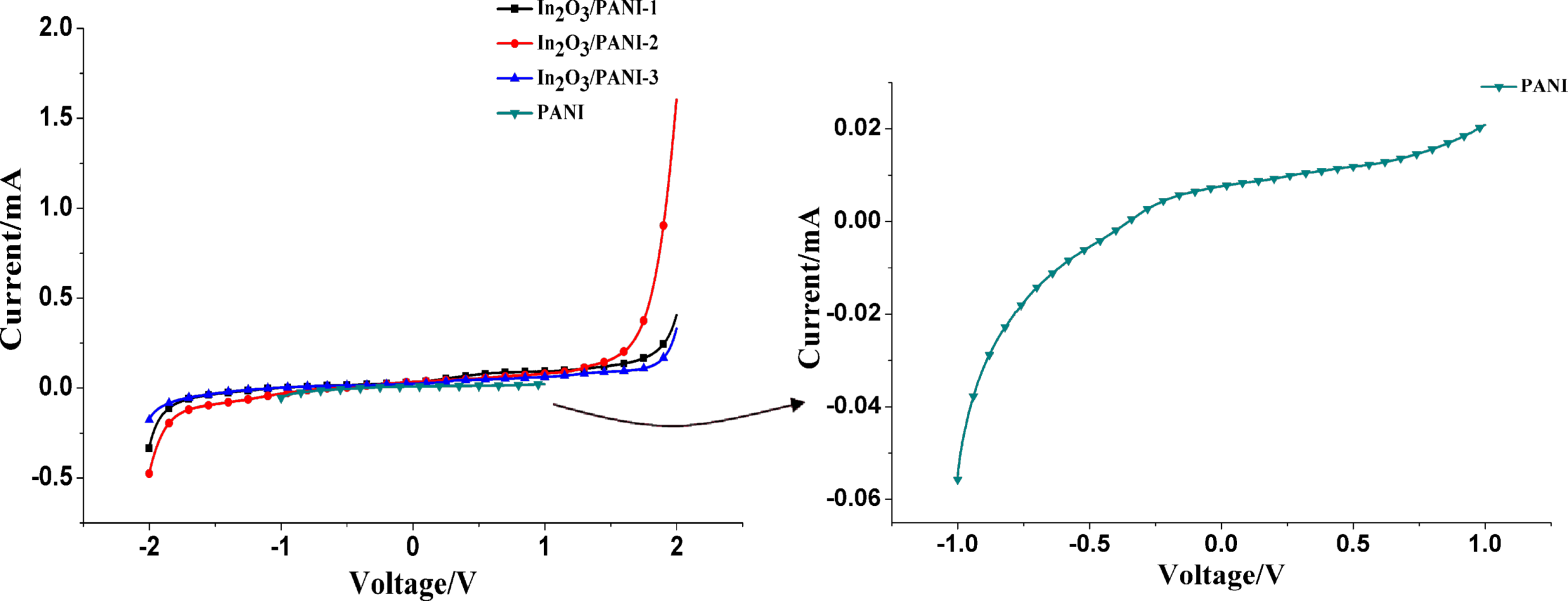
Current–Voltage (*I*–*V*) characteristics of pure PANI and In_2_O_3_/PANI nanofibers.

Current–voltage (*I*–*V*) measurements of PANI, In_2_O_3_/PANI-1, In_2_O_3_/PANI-2 and In_2_O_3_/PANI-3 nanofibers were carried out at room temperature. As shown in Figure 4, the *I*–*V* characteristics of all In_2_O_3_/PANI nanofibers clearly exhibit a nonlinear behavior and it can be observed the rectifying behavior in Figure 4, which might result from the p–n junction between the p-type PANI and n-type In_2_O_3_ [28–29]. It can be observed that the current of In_2_O_3_/PANI showed exponential rise at low voltage and then almost linear rise at high voltages. But for pure PANI, the current showed nearly linear behavior in the forward region, which is attributed to raidply forming polarons and bipolarons in PANI. As some researches mentioned [28–30], the ohmic behavior in this case was related to the formation of an ohmic contact between PANI and In_2_O_3_. Compared with pure PANI, the In_2_O_3_/PANI nanofibers reach a higher current due to the smaller width of the depletion layer between PANI and In_2_O_3_. Moreover, the addition of PANI reduced the width of the depletion layer at the interface and was helpful to form a typical ohmic system [31]. Thus, it can be confirmed that a p–n junction between PANI and In_2_O_3_ had been formed.

### Gas sensing properties

**Figure 5:**
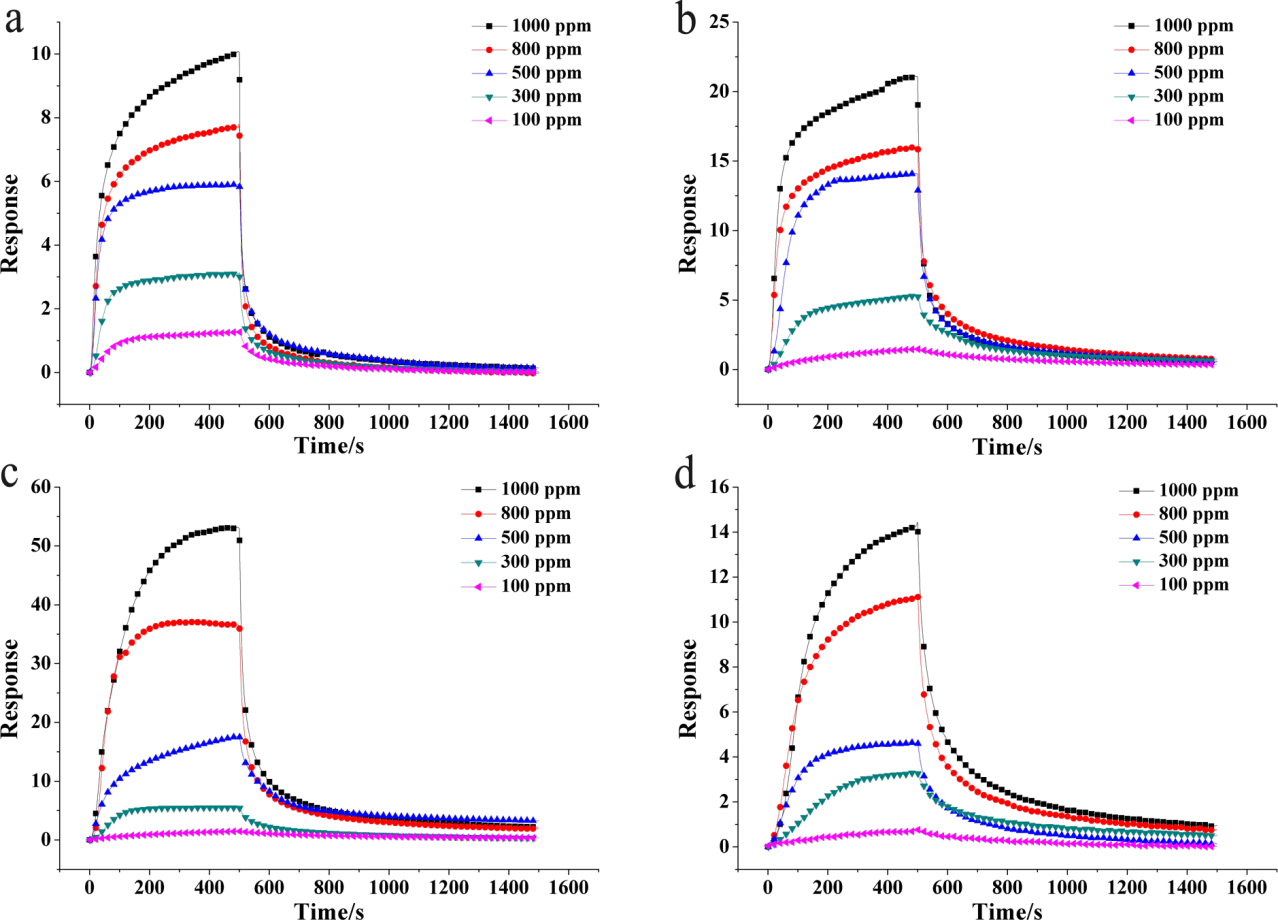
Dynamic response of sensors based on (a) pure PANI, (b) In_2_O_3_/PANI nanofibers-1, (c) In_2_O_3_/PANI nanofibers-2 and (d) In_2_O_3_/PANI nanofibers-3 towards 100–1000 ppm NH_3_ at room temperature.

To study the ammonia sensing behavior of the sensors with different ratios of In_2_O_3_ to aniline, the dynamic response of the sensors based on pure PANI and In_2_O_3_/PANI nanofibers towards different NH_3_ concentrations ranging from 100 to 1000 ppm at room temperature were investigated. As exhibited in Figure 5, it can be seen that the trends of the response and recovery were consistent among the pure PANI and the three In_2_O_3_/PANI nanofibers sensors. The In_2_O_3_/PANI-1 and In_2_O_3_/PANI-2 nanofibers always show a higher response value than pure PANI at the same concentration of NH_3_.

**Figure 6:**
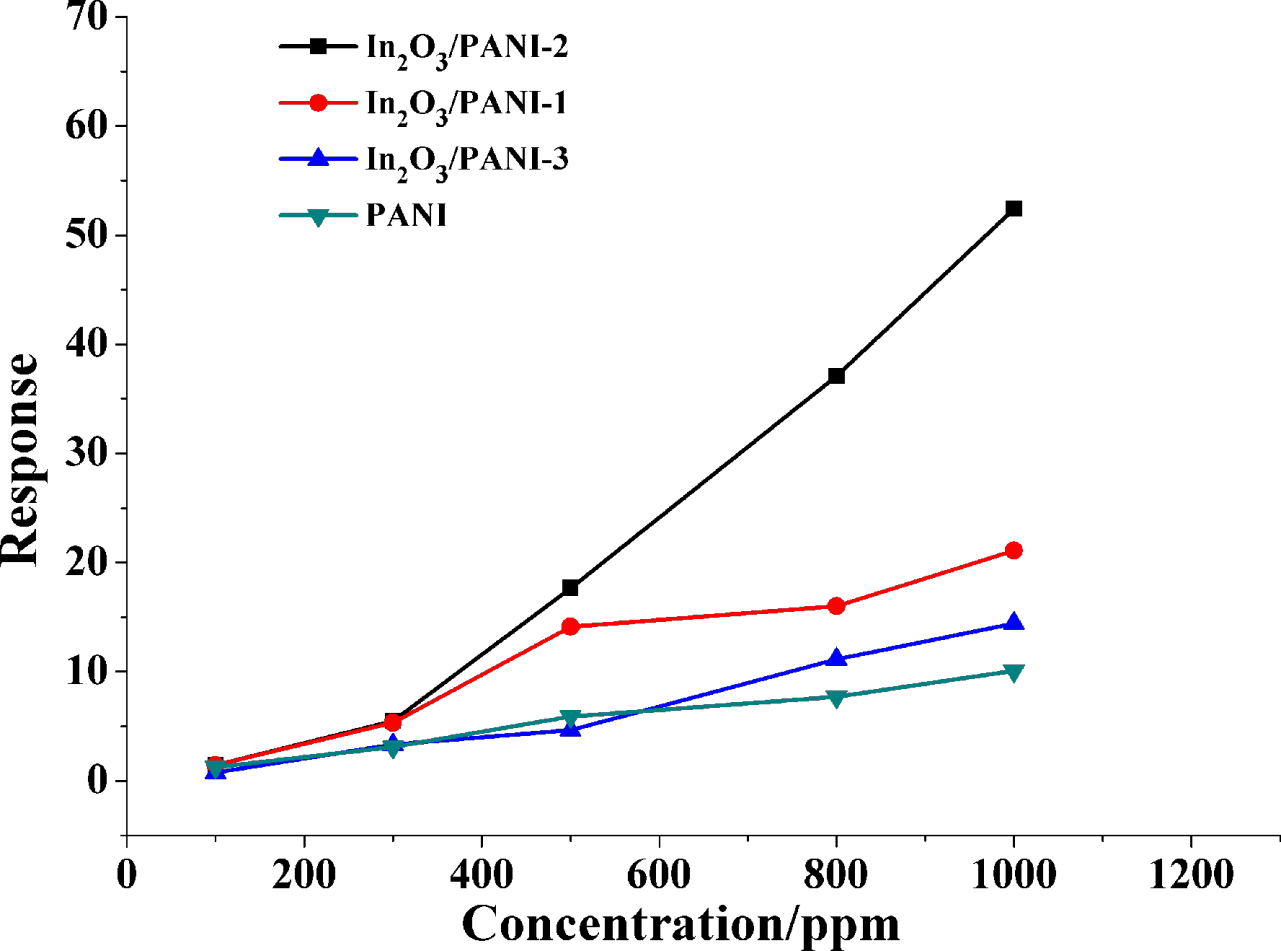
The response values of pure PANI and three In_2_O_3_/ PANI nanofibers sensors to different concentration of NH_3_ at room temperature.

The response values of pure PANI and these three In_2_O_3_/PANI nanofibers sensors to different concentrations of NH_3_ are displayed in Figure 6. It can be found that the response values increased with the growth of gas concentration. The responses of pure PANI to 100 ppm, 300 ppm, 500 ppm, 800 ppm, 1000 ppm NH_3_ were 1.26, 3.11, 5.91, 7.71, 10.09, respectively. The responses of In_2_O_3_/PANI-1 nanofibers sensor were 1.48, 5.32, 14.11, 16.00 and 21.12. For In_2_O_3_/PANI-2 nanofibers sensor, the responses were 1.48, 5.48, 17.67, 36.57 and 53.20, respectively. While for the In_2_O_3_/PANI-3 sensor, the values were only 0.76, 3.32, 4.64, 11.15 and 14.43. It was observed that at low concentrations of NH_3_, the responses of these sensors were similar. But with increasing NH_3_ concentrations, it was very clear that the responses of In_2_O_3_/PANI gas sensors were much higher than pure PANI. The response of In_2_O_3_/PANI-2 exhibited the highest value. The response of In_2_O_3_/PANI-2 to 1000 ppm was about twice as large as that of In_2_O_3_/PANI-1. When the weight ratio of In_2_O_3_ to aniline was raised to 1:4 (In_2_O_3_/PANI-3), the responses to NH_3_ decreased.

These results revealed that the mass ratio of In_2_O_3_ to aniline had an obvious influence on the NH_3_ sensing performance of the composite nanofibers. Comparing the sensitivity of three In_2_O_3_/PANI nanofibers sensors, it can be found that the In_2_O_3_/PANI-2 nanofibers sensor delivers the best performance. Therefore, In_2_O_3_/PANI-2 was selected to further investigate the sensing properties.

**Figure 7:**
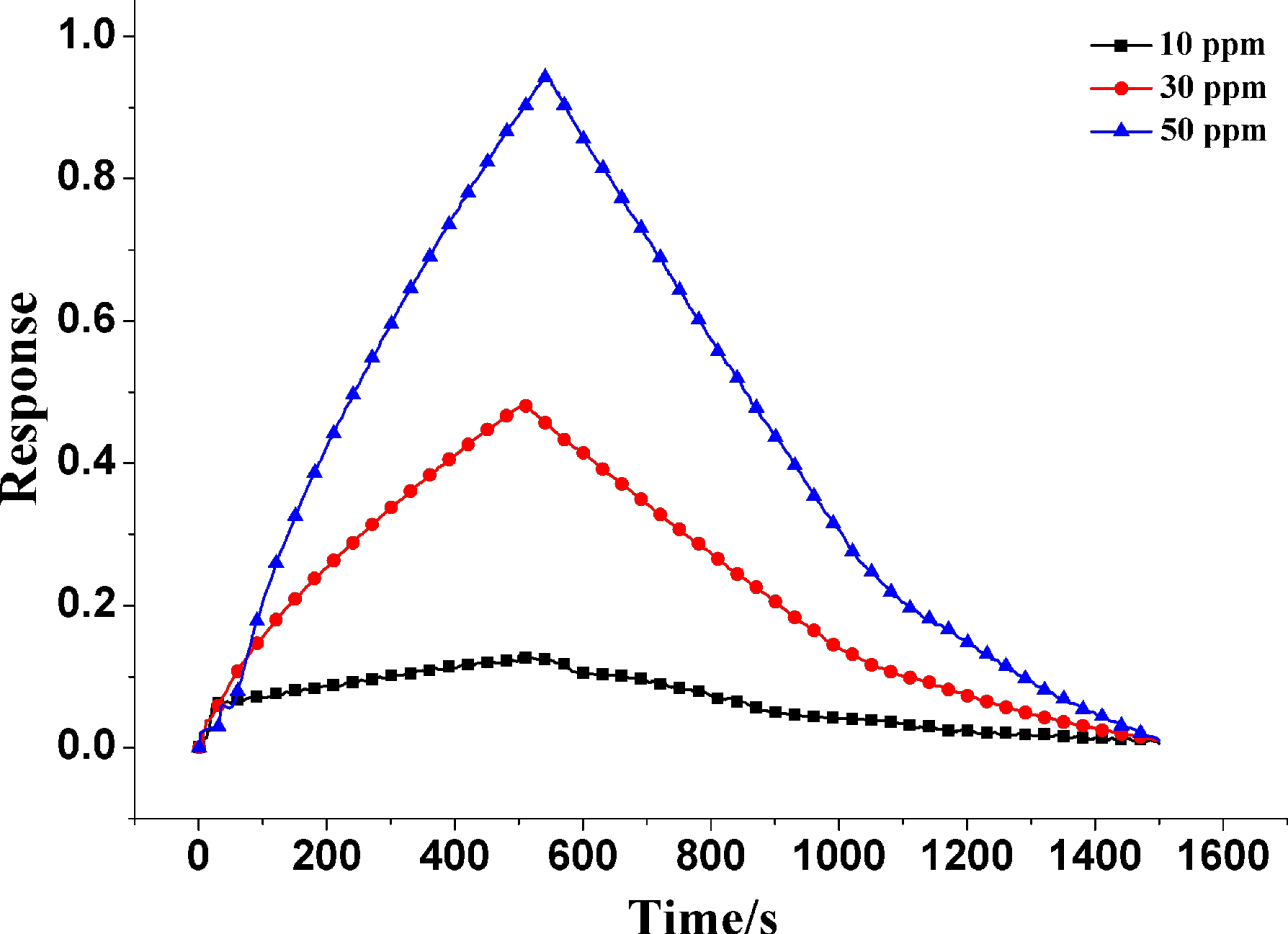
Dynamic response of In_2_O_3_/ PANI-2 sensor towards 50 ppm, 30 ppm and 10 ppm NH_3_ at room temperature.

As mentioned in the Introduction section, 50 ppm NH_3_ will cause harm to human health. Accordingly, the response of the In_2_O_3_/PANI-2 sensor to 50 ppm, 30 ppm and 10 ppm were investigated. As shown in Figure 7, the response of the In_2_O_3_/PANI-2 sensor to low concentration (10–50 ppm) NH_3_ was 0.12, 0.48 and 0.94, respectively. Thus it can be seen the In_2_O_3_/PANI-2 sensor had good response performance towards low concentrations of NH_3_.

**Figure 8:**
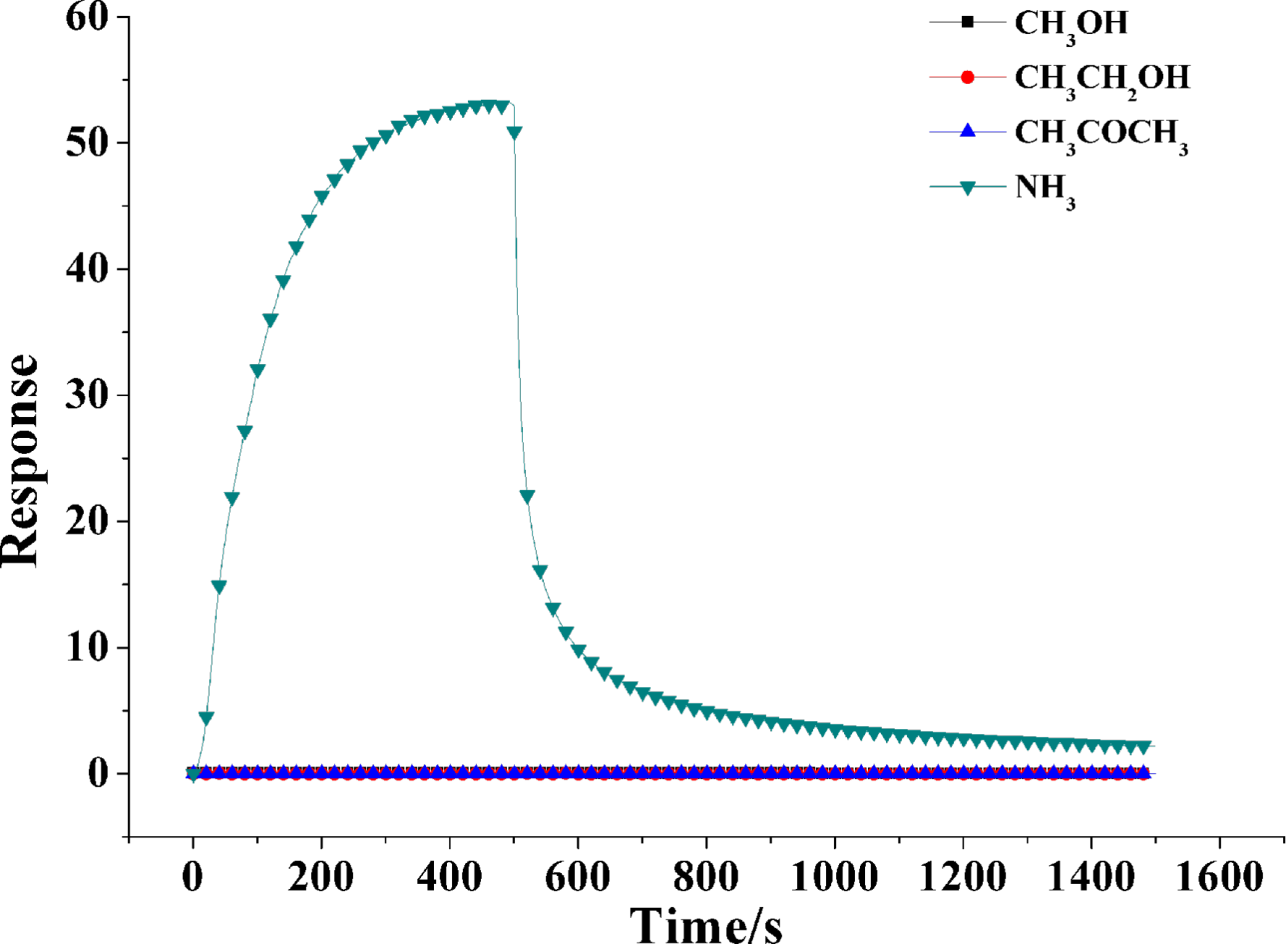
Cross-response curves of In_2_O_3_/PANI-2 nanofibers sensor to 1000 ppm methanol, ethanol, acetone and ammonia.

The cross-response test was used to evaluate the selectivity of In_2_O_3_/PANI-2 nanofibers sensor. Figure 8 shows the dynamic response of In_2_O_3_/PANI-2 nanofibers sensor to methanol, ethanol, acetone and ammonia at a concentration of 1000 ppm. It is obvious that the In_2_O_3_/PANI-2 nanofibers sensor was almost insensitive to methanol, ethanol and acetone vapors. According to the test results, it can be concluded that In_2_O_3_/PANI-2 nanofibers sensor exhibited unique selectivity to ammonia. A possible mechanism for the selectivity to NH_3_ is the chemisorption of NH_3_ on PANI in In_2_O_3_/PANI-2 forming ammonium [32]. Besides, the different gases show different electron affinity values [33], and the varying sensitivity of In_2_O_3_/PANI-2 nanofibers to different gases may be explained by this.

**Figure 9:**
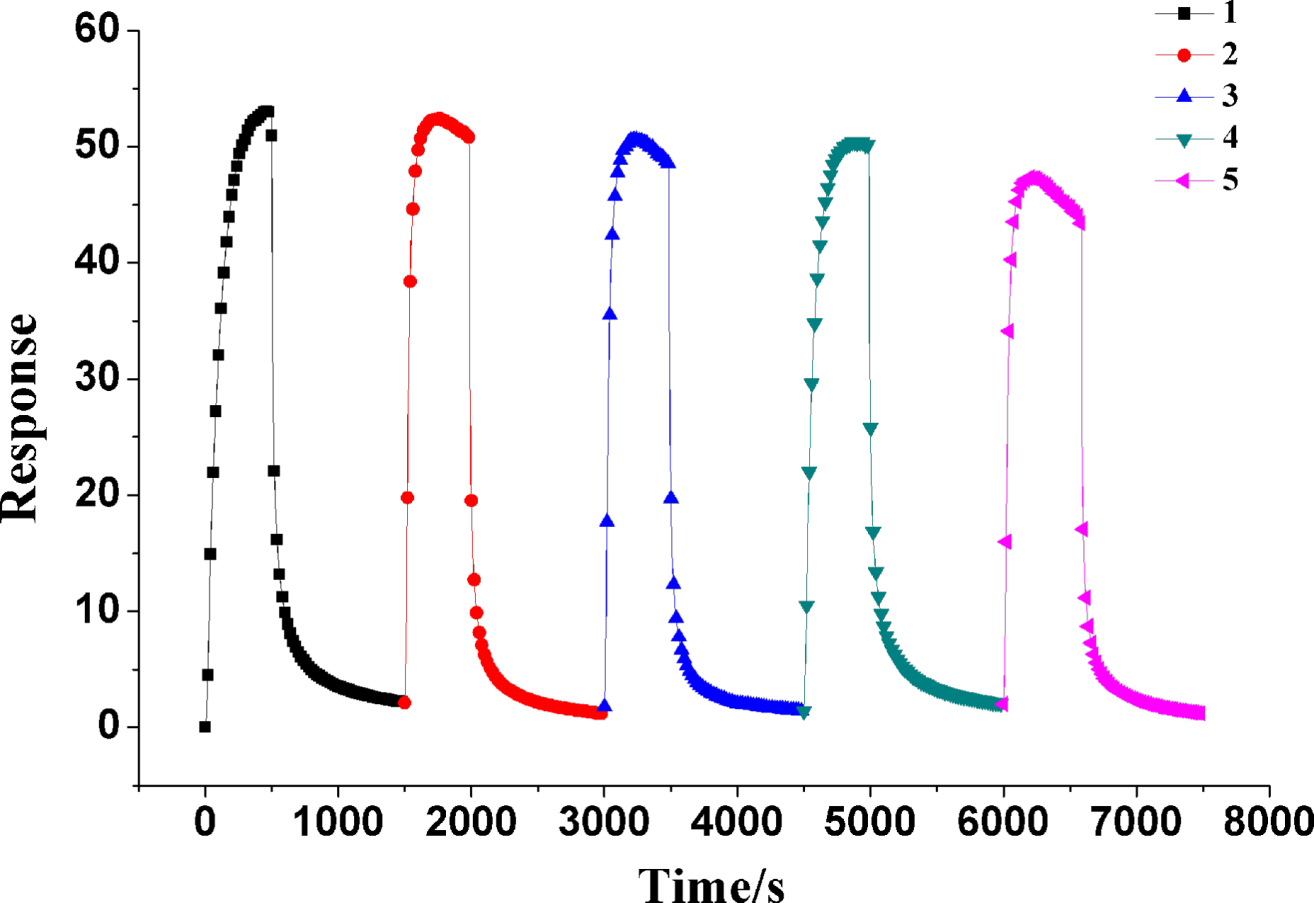
Sensing repeatability and reversibility of In_2_O_3_/PANI-2 nanofibers sensor to 1000 ppm NH_3_ vapor.

In_2_O_3_/PANI-2 nanofibers sensor was exposed to 1000 ppm ammonia for five times to investigate the repeatability and reversibility. As shown in Figure 9, the recovery of the In_2_O_3_/PANI-2 nanofibers sensor could not fully return to the initial state, and there was a baseline drift of 4% after the first exposure to NH_3_. This bias was smaller than the results in other reports [34–36]. On the other hand, the response of this sensor slightly decreased with the increasing number of tests. The final response reached 47.42, which was about 89% of the first test. Hence, the In_2_O_3_/PANI-2 nanofibers sensor showed good repeatability and reversibility.

### Gas sensing mechanism

It is well known that the chemical sensors are composed of two parts, an active part and a transduction part, whose function is sensitive to gas analytes and produces a signal that is related to the concentration [19]. In this study, PANI acted as an active element which can react with NH_3_ resulting in the transformation of PANI from emeraldine salt to emeraldine base by dedoping. The reaction between PANI and NH_3_ can be described as follows:


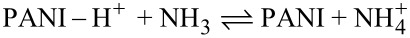


The absorption of NH_3_ caused the deprotonation of the N–H^+^ site of the emeraldine salt, leading to a significantly increased resistance [37–38]. When this reaction reached equilibrium in NH_3_ atmosphere, the resistance of the PANI-based sensor maintained a constant value. When the sensor was exposed to air, NH_3_ is volatilized and the resistance of the PANI composite nanofibers is reduced. Therefore, due to the unique mechanism, PANI (emeraldine salt) based sensors exhibited a great selectivity to NH_3_.

**Figure 10:**
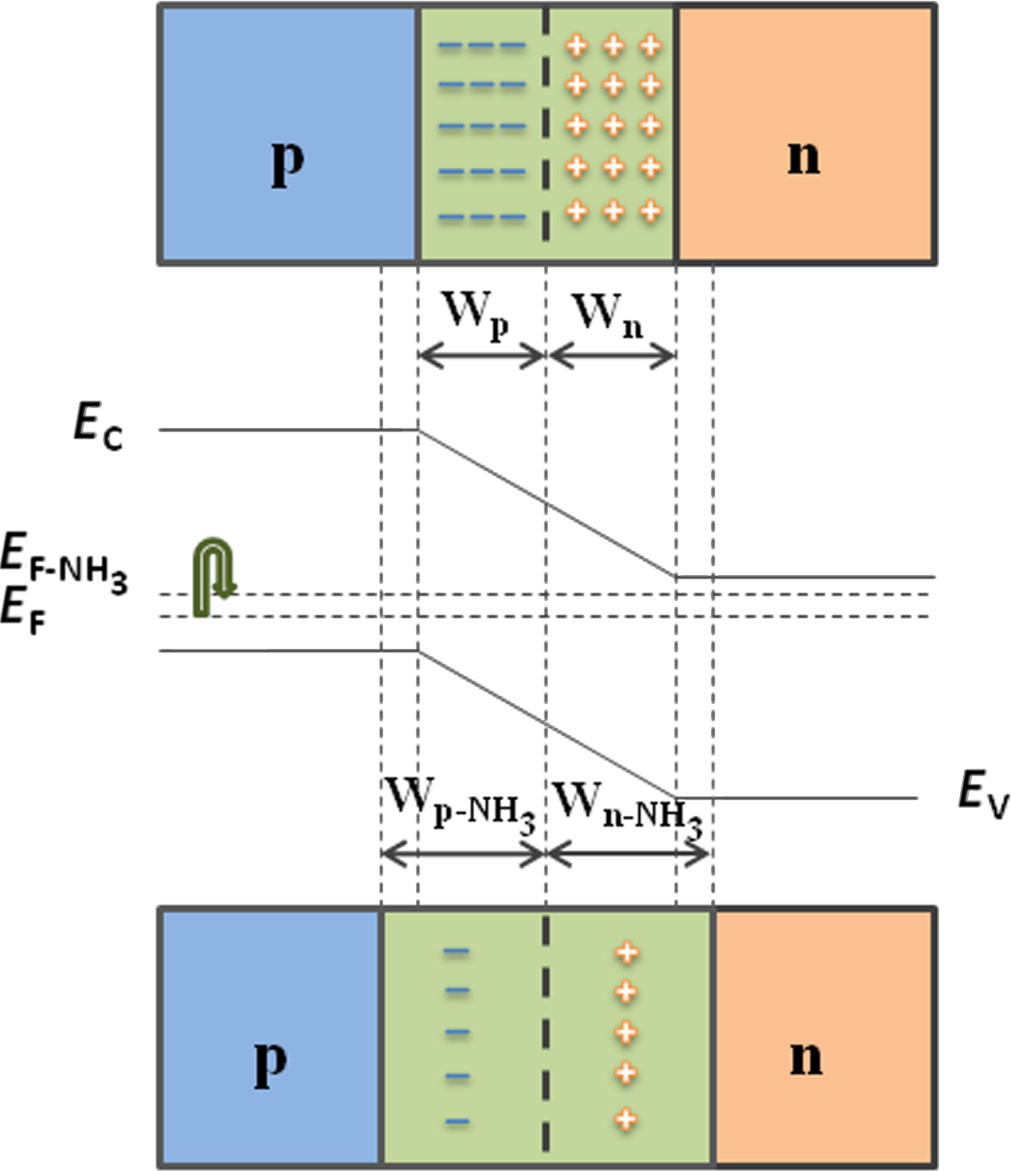
Schematic of p–n junction of In_2_O_3_/PANI nanofibers and its potential energy barrier change when exposed to NH_3_.

As the *I*–*V* characteristics show, it is confirmed that p–n heterojunctions had been formed between PANI and In_2_O_3_ nanofibers, in which PANI is a p-type semiconductor and In_2_O_3_ nanofibers presents as an n-type semiconductor [21,27,34]. The changes of the depletion layer of the p–n heterojuction are shown in Figure 10. The width of the depletion section is related to the doping concentration [39]. With low concentration doping, it needs a sufficiently thick depletion layer to provide impurity atoms to build an internal field. Accordingly, on exposure to NH_3_, the protons from PANI are transferred to the NH_3_ molecules, which results in a widening of the depletion layer in the PANI section [40]. Simultaneously, the variation of the PANI region width would have effects on the width of the In_2_O_3_ region and on the p–n junction. The electrons of In_2_O_3_ and holes of PANI move in opposite directions until the new Fermi level (*E*_F-NH3_) reaches equilibrium. In this process, the electron transfer between the n-type In_2_O_3_ and p-type PANI is obstructed due to the potential barrier. Thus the depletion layer between PANI and In_2_O_3_ becomes wider and the resistance of the material increases [27,41–42]. According to the response definition (*S* = (*R*_i_ − *R*_0_)/*R*_0_), the increase in resistance attributed to the p–n junction increases the sensitivity of composite nanofibers sensors. In addition, the sensitivity of the composite gas sensor materials is also connected to the mass ratio of In_2_O_3_ and aniline.

Because of the smaller amount of polyaniline, which acts as active material in this composite system, the In_2_O_3_/PANI-1 gas sensor showed a lower sensitivity. However, for the In_2_O_3_/PANI-3 gas sensor, the external surface of In_2_O_3_ nanofibers was coated with excess polyaniline, yielding a gas sensing perfomance similar to that of pure PANI. Even the response of In_2_O_3_/PANI-3 to 100 ppm and 500 ppm NH_3_ was less than that of pure PANI. The p–n junction of In_2_O_3_ and PANI in In_2_O_3_/PANI-3 does not work efficiently. In general, the characteristic of the gas sensitive material response was discrete instead of ideally linear [34,36,43]. For In_2_O_3_/PANI-3 and pure PANI, the characteristic responses were similar. Therefore, it was possible that the response of In_2_O_3_/PANI-3 were less than that of pure PANI, especially towards lower concentrations of NH_3_. In this study, when the mass ratio of In_2_O_3_ to aniline was 1:2, the gas sensor material exhibited optimum performance in detecting NH_3_.

## Conclusion

In_2_O_3_/PANI nanofibers with reliable sensing properties towards NH_3_ were synthesized by electrospinning, calcination and in situ polymerization. The gas sensors based on In_2_O_3_/PANI nanofibers exhibited a higher sensitivity than pure PANI. The In_2_O_3_/PANI-2 nanofiber sensor exhibited the best sensitivity to NH_3_ vapor at room temperature, and this sensor was further investigated for its selectivity by interfering with methanol, ethanol and acetone vapors. The results indicated that the In_2_O_3_/PANI-2 nanofiber sensor had excellent selectivity, good repeatability and reversibility. The enhancement of gas sensing performance of In_2_O_3_/PANI nanofiber sensor may be attributed to formation of a p–n junction between In_2_O_3_ and PANI, which existence is confirmed by the *I*–*V* characteristics.

## References

[1] Tang Y-L, Li Z-J, Ma J-Y, Su H-Q, Guo Y-J, Wang L, Du B, Chen J-J, Zhou W, Yu Q-K (2014). J Hazard Mater.

[2] Wu Z, Chen X, Zhu S, Zhou Z, Yao Y, Quan W, Liu B (2013). Sens Actuators, B.

[3] Na K, Song C, Switzer C, Cocker D R (2007). Environ Sci Technol.

[4] Jabłońska M, Palkovits R (2016). Appl Catal, B.

[5] Swotinsky R B, Chase K H (1990). Am J Ind Med.

[6] Park S-J, Jin S-Y (2005). J Colloid Interface Sci.

[7] Yoo K-P, Kwon K-H, Mi N-K, Lee M J, Lee C J (2009). Sens Actuators, B.

[8] Li C, Zhang D, Lei B, Han S, Liu X, Zhou C (2003). J Phys Chem B.

[9] Biskupski D, Herbig B, Schottner G, Moos R (2011). Sens Actuators, B.

[10] Shahabuddin M, Sharma A, Kumar J, Tomar M, Umar A, Gupta V (2014). Sens Actuators, B.

[11] Renganathan B, Sastikumar D, Gobi G, Yogamalar N R, Bose A C (2011). Opt Laser Technol.

[12] Nguyen D D, Dang D V, Nguyen D C (2015). Adv Nat Sci: Nanosci Nanotechnol.

[13] Zhang D, Liu Z, Li C, Tang T, Liu X, Han S, Lei B, Zhou C (2004). Nano Lett.

[14] Miller D R, Akbar S A, Morris P A (2014). Sens Actuators, B.

[15] Zhan Z, Jiang D, Xu J (2005). Mater Chem Phys.

[16] Liang X, Jin G, Liu F, Zhang X, An S, Ma J, Lu G (2015). Ceram Int.

[17] Li Z, Fan Y, Zhan J (2010). Eur J Inorg Chem.

[18] Li H, Liu Y, Luo L, Tan Y, Zhang Q, Li K (2016). Mater Sci Eng, C.

[19] Fratoddi I, Venditti I, Cametti C, Russo M V (2015). Sens Actuators, B.

[20] Lakard B, Carquigny S, Segut O, Patios T, Lakard S (2015). Metals (Basel, Switz).

[21] Gong J, Li Y, Hu Z, Zhou Z, Deng Y (2010). J Phys Chem C.

[22] Timmer B, Olthuis W, van den Berg A (2005). Sens Actuators, B.

[23] Pawar S G, Chougule M A, Patil S L, Raut B T, Godse P R, Sen S, Patil V B (2011). IEEE Sens J.

[24] Nasirian S, Milani M H (2015). Appl Surf Sci.

[25] Sadek A Z, Wlodarski W, Shin K, Kaner R B, Kalantar-zadeh K (2008). Synth Met.

[26] Betty C A, Choudhury S, Arora S (2015). Sens Actuators, B.

[27] Pang Z, Fu J, Luo L, Huang F, Wei Q (2014). Colloids Surf, A.

[28] Abaci S, Nessark B, Riahi F (2014). Ionics.

[29] Ameen S, Shaheer A M, Ansari S G, Yang O-B, Shin H-S (2009). Superlattices Microstruct.

[30] Li Y, Gong J, McCune M, He G, Deng Y (2010). Synth Met.

[31] Pandey S S, Rikitake K, Takashima W, Kaneto K (2003). Curr Appl Phys.

[32] Tai H, Xu X, Ye Z, Liu C, Xie G, Jiang Y (2015). Chem Phys Lett.

[33] Pawar S G, Chougule M A, Shashwati S, Patil V B (2012). J Appl Polym Sci.

[34] Wang L, Huang H, Xiao S, Cai D, Liu Y, Liu B, Wang D, Wang C, Li H, Wang Y (2014). ACS Appl Mater Interfaces.

[35] Matsuguchi M, Asahi T (2011). Sens Actuators, B.

[36] Joulazadeh M, Navarchian A H (2015). Synth Met.

[37] Zhang Y, Kim J J, Chen D, Tuller L H, Rutledge C C (2014). Adv Funct Mater.

[38] Srinives S, Sarkar T, Mulchandani A (2013). Electroanalysis.

[39] Mane A T, Navale S T, Patil V B (2015). Org Electron.

[40] Bai S, Tian Y, Cui M, Sun J, Tian Y, Luo R, Chen A, Li D (2016). Sens Actuators, B.

[41] Xu H, Ju D, Li W, Gong H, Zhang J, Wang J, Cao B (2016). Sens Actuators, B.

[42] Bai S, Ma Y, Luo R, Chen A, Li D (2016). RSC Adv.

[43] Li Y, Ban H, Yang M (2016). Sens Actuators, B.

